# Supplementary material for: Decoding mechanism of action and sensitivity to drug candidates from integrated transcriptome and chromatin state
Source: eLife. 2022 Aug 31;11:e78012. doi: 10.7554/eLife.78012 (PMC9433094; doi:10.7554/eLife.78012)
Supplement: Supplementary file 10. [file elife-78012-supp10.pdf]

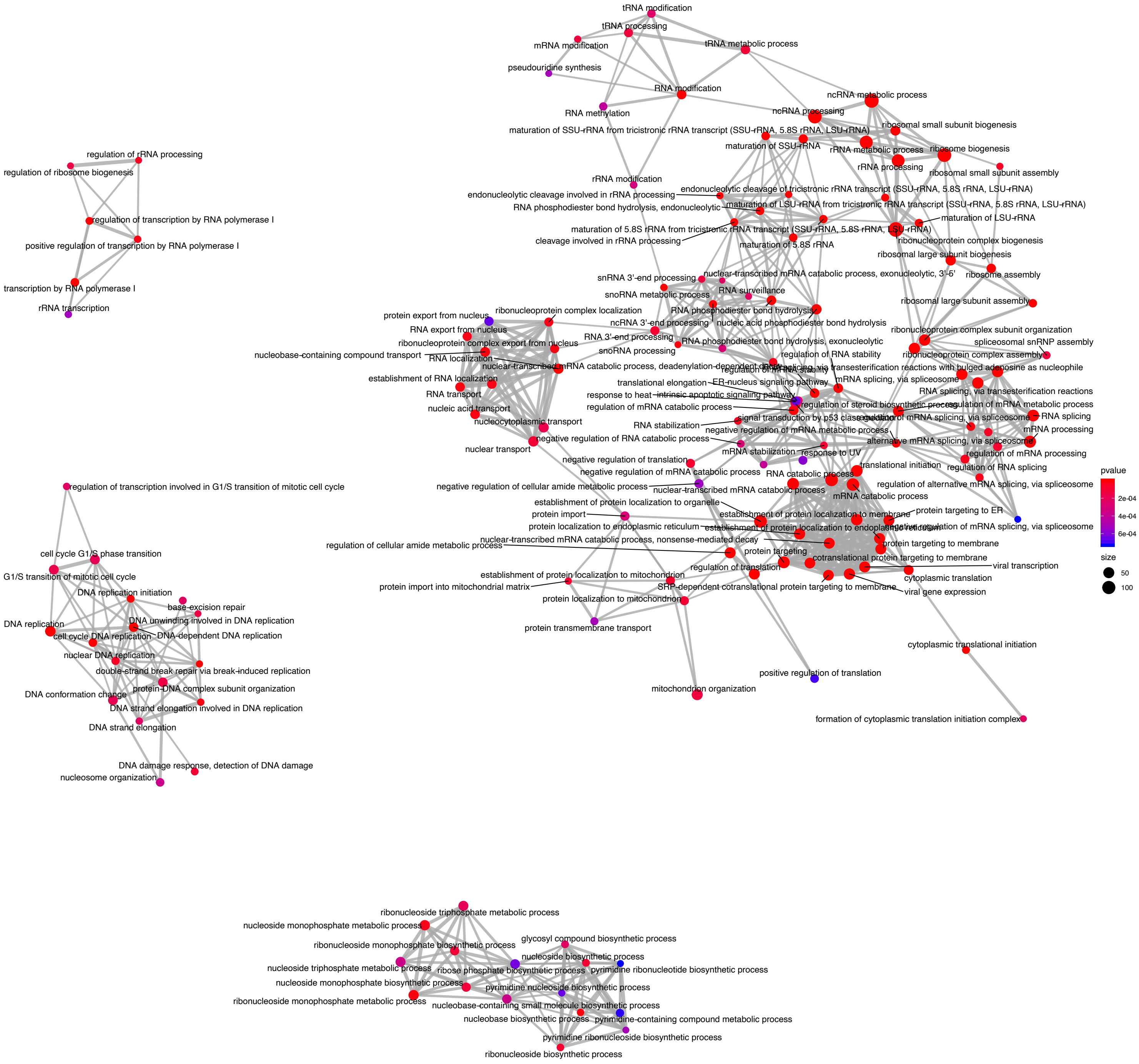

Map of the most significant GO terms (150 nodes) enriched in the union of DE genes upregulated in BxPC-3 cells after treatment (DE genes identified after 6 h and 12 h of treatment with both **M** and **B**).

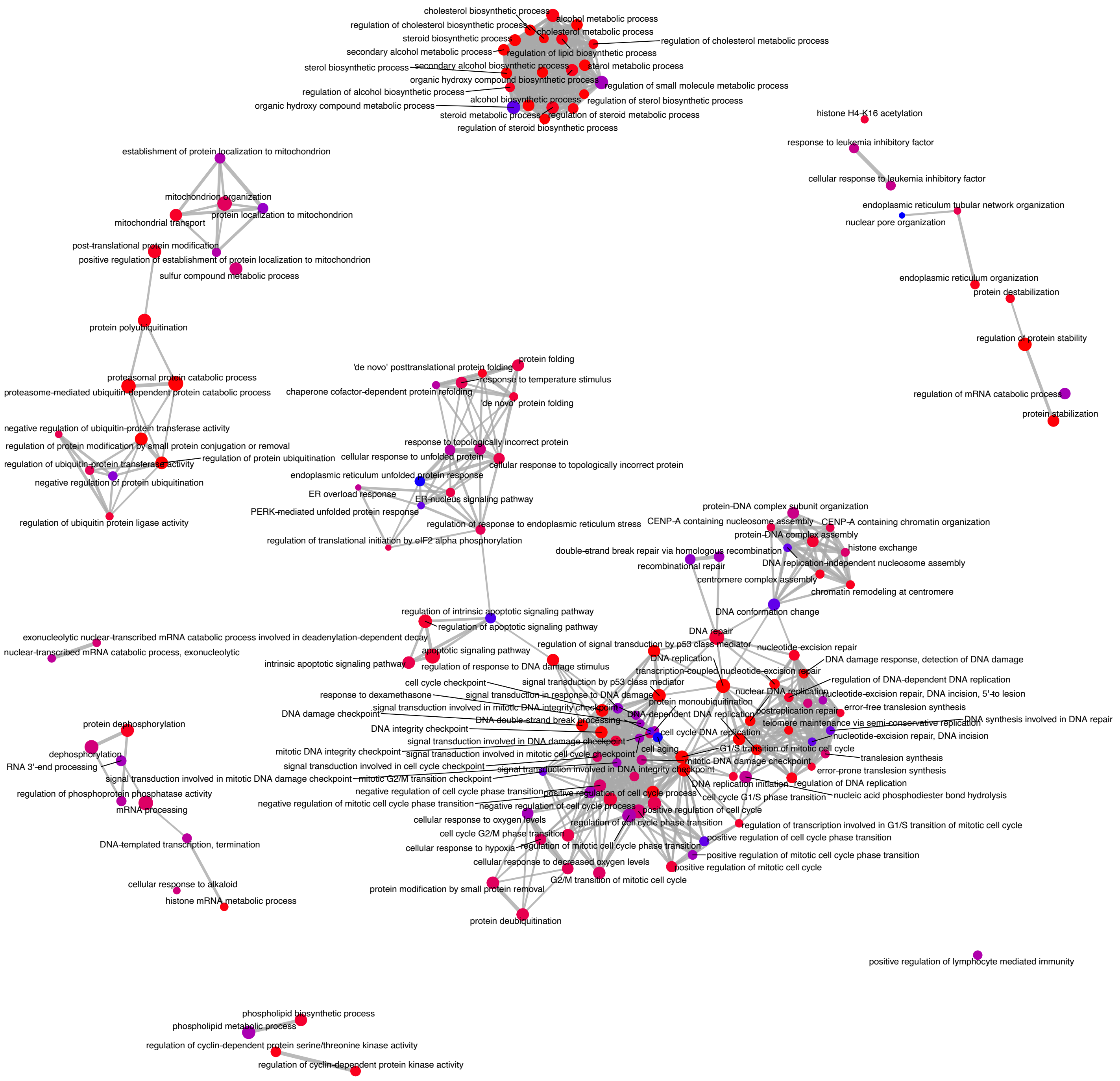

Map of the most significant GO terms (150 nodes) enriched in the union of DE genes upregulated in HCT-15 cells after treatment (DE genes identified after 6 h and 12 h of treatment with both **M** and **B**).

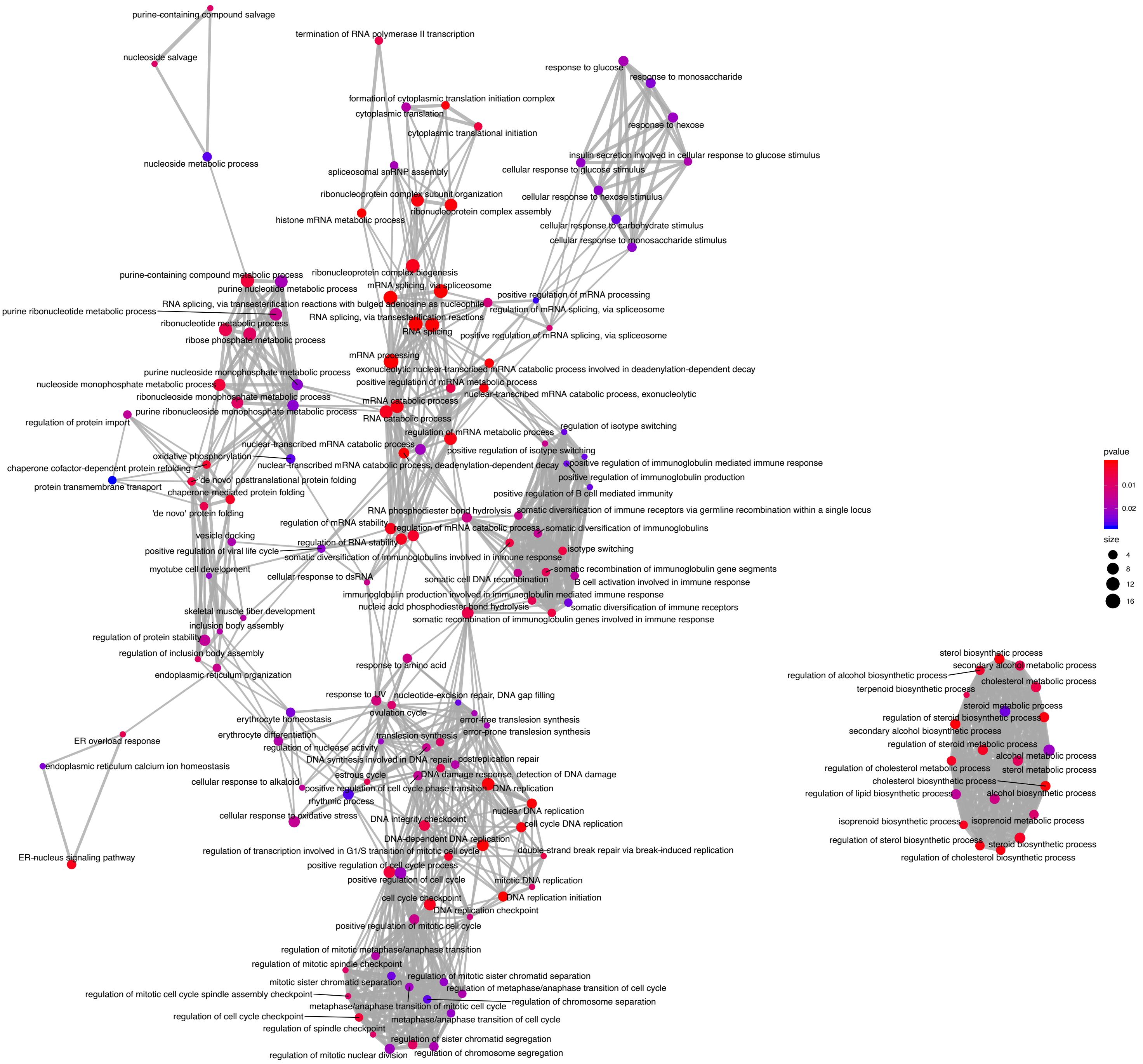

Map of the most significant GO terms (150 nodes) enriched in the union of shared DE genes upregulated in BxPC-3 and HCT-15 cells after treatment (DE genes identified after 6 h and 12 h of treatment with both **M** and **B**).

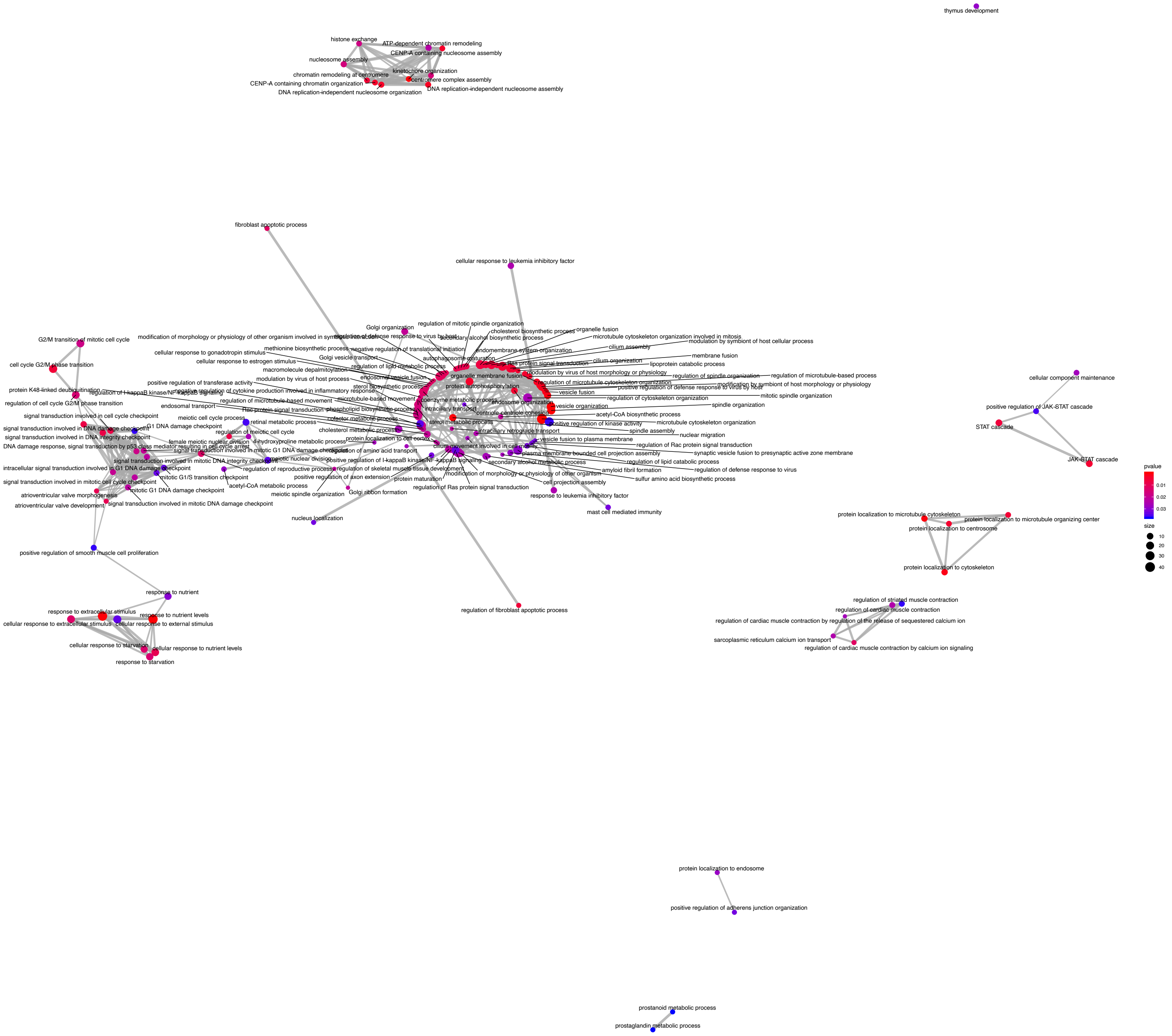

Map of the most significant GO terms (150 nodes) enriched in the union of DE genes downregulated in BxPC-3 cells after treatment (DE genes identified after 6 h and 12 h of treatment with both **M** and **B**).

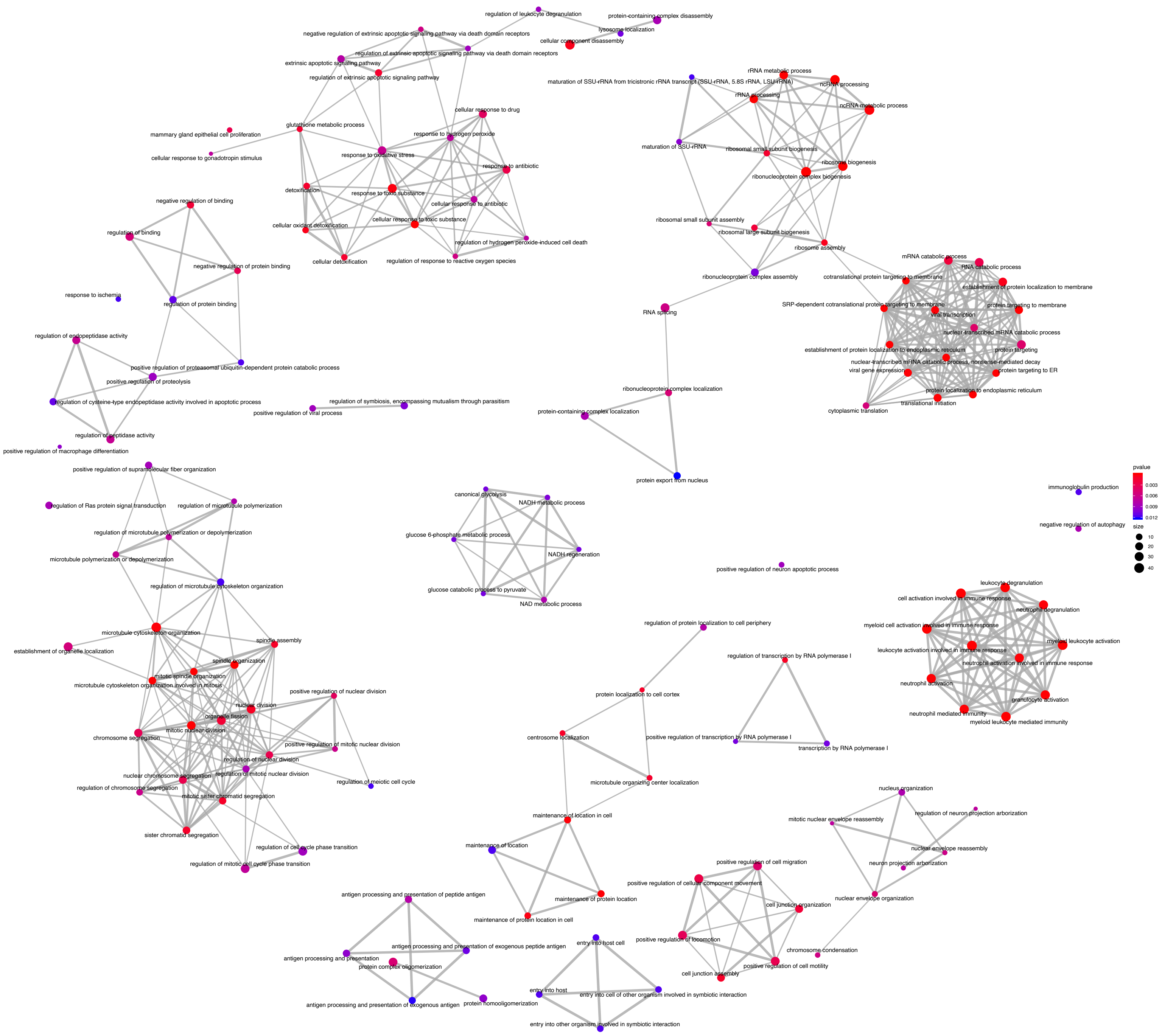

Map of the most significant GO terms (150 nodes) enriched in the union of DE genes downregulated in HCT-15 cells after treatment (DE genes identified after 6 h and 12 h of treatment with both **M** and **B**).

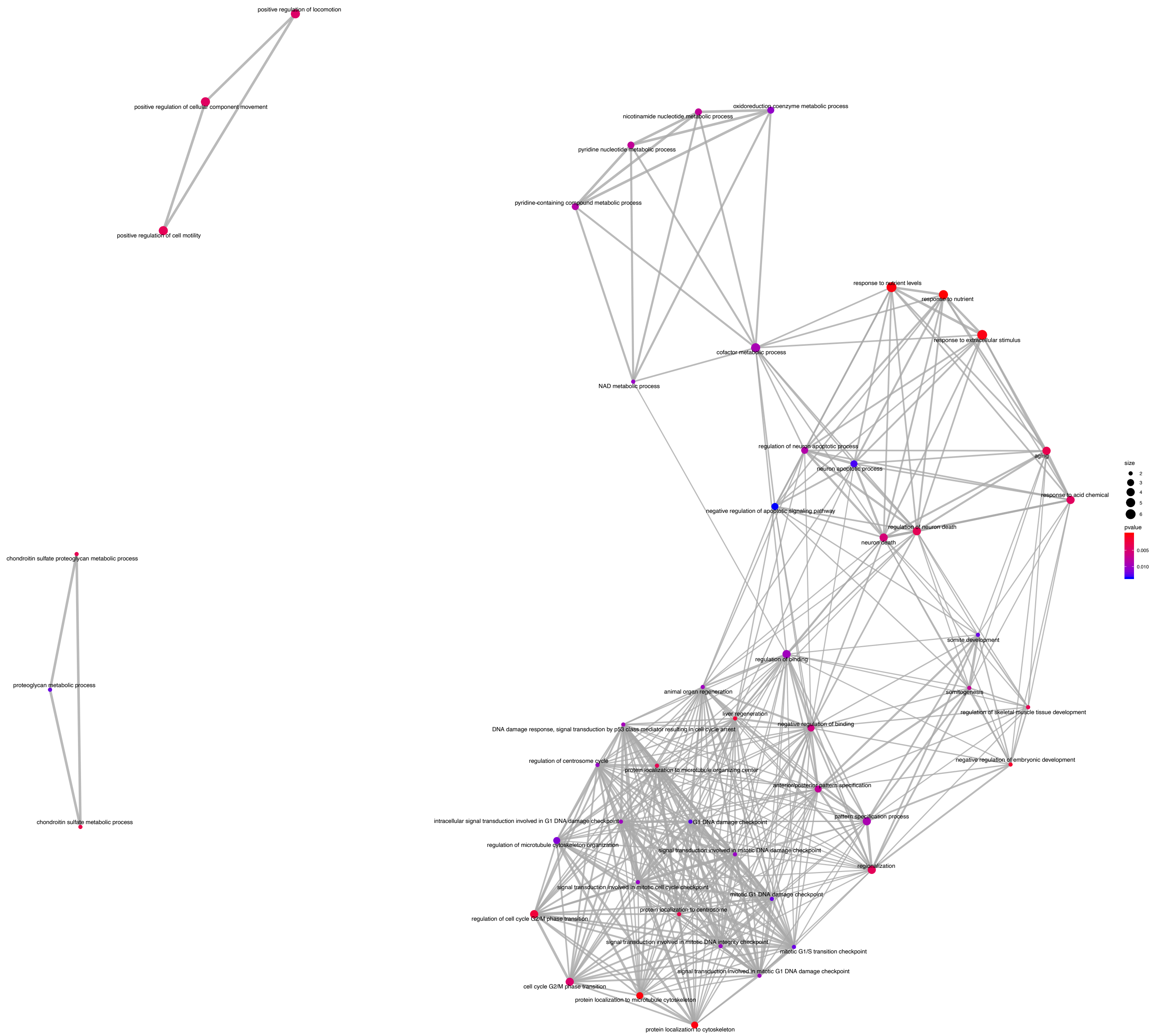

Map of the most significant GO terms (50 nodes) enriched in the union of shared DE genes downregulated in BxPC-3 and HCT-15 cells after treatment (DE genes identified after 6 h and 12 h of treatment with both **M** and **B**).
