# Supplementary material for: Decoding mechanism of action and sensitivity to drug candidates from integrated transcriptome and chromatin state
Source: eLife. 2022 Aug 31;11:e78012. doi: 10.7554/eLife.78012 (PMC9433094; doi:10.7554/eLife.78012)
Supplement: Transparent reporting form [file elife-78012-transrepform1.docx]

***eLife’s* transparent reporting form**

We encourage authors to provide detailed information *within their submission* to facilitate the interpretation and replication of experiments. Authors can upload supporting documentation to indicate the use of appropriate reporting guidelines for health-related research (see [EQUATOR Network](http://www.equator-network.org/%20)), life science research (see the [BioSharing Information Resource](https://biosharing.org/)), or the [ARRIVE guidelines](http://www.plosbiology.org/article/info:doi/10.1371/journal.pbio.1000412) for reporting work involving animal research. Where applicable, authors should refer to any relevant reporting standards documents in this form.

If you have any questions, please consult our Journal Policies and/or contact us: [editorial@elifesciences.org](mailto:editorial@elifesciences.org).

**Sample-size estimation**

- You should state whether an appropriate sample size was computed when the study was being designed
- You should state the statistical method of sample size computation and any required assumptions
- If no explicit power analysis was used, you should describe how you decided what sample (replicate) size (number) to use

Please outline where this information can be found within the submission (e.g., sections or figure legends), or explain why this information doesn’t apply to your submission:

Such information is specified in the *Statistics and Reproducibility* section.

**Replicates**

- You should report how often each experiment was performed
- You should include a definition of biological versus technical replication
- The data obtained should be provided and sufficient information should be provided to indicate the number of independent biological and/or technical replicates
- If you encountered any outliers, you should describe how these were handled
- Criteria for exclusion/inclusion of data should be clearly stated
- High-throughput sequence data should be uploaded before submission, with a private link for reviewers provided (these are available from both GEO and ArrayExpress)

Please outline where this information can be found within the submission (e.g., sections or figure legends), or explain why this information doesn’t apply to your submission:

The required information about replicates and sample size is reported in the *Methods* and *Data Availability* sections, as well as figure legends where applicable.

**Statistical reporting**

- Statistical analysis methods should be described and justified
- Raw data should be presented in figures whenever informative to do so (typically when N per group is less than 10)
- For each experiment, you should identify the statistical tests used, exact values of N, definitions of center, methods of multiple test correction, and dispersion and precision measures (e.g., mean, median, SD, SEM, confidence intervals; and, for the major substantive results, a measure of effect size (e.g., Pearson's r, Cohen's d)
- Report exact p-values wherever possible alongside the summary statistics and 95% confidence intervals. These should be reported for all key questions and not only when the p-value is less than 0.05.

Please outline where this information can be found within the submission (e.g., sections or figure legends), or explain why this information doesn’t apply to your submission:

The required statistical information is reported in the *Methods/Statistics and Reproducibility* sections, as well as figure legends where applicable.

(For large datasets, or papers with a very large number of statistical tests, you may upload a single table file with tests, Ns, etc., with reference to sections in the manuscript.)

**Group allocation**

- Indicate how samples were allocated into experimental groups (in the case of clinical studies, please specify allocation to treatment method); if randomization was used, please also state if restricted randomization was applied
- Indicate if masking was used during group allocation, data collection and/or data analysis

Please outline where this information can be found within the submission (e.g., sections or figure legends), or explain why this information doesn’t apply to your submission:

Not applied

**Additional data files (“source data”)**

- We encourage you to upload relevant additional data files, such as numerical data that are represented as a graph in a figure, or as a summary table
- Where provided, these should be in the most useful format, and they can be uploaded as “Source data” files linked to a main figure or table
- Include model definition files including the full list of parameters used
- Include code used for data analysis (e.g., R, MatLab)
- Avoid stating that data files are “available upon request”

Please indicate the figures or tables for which source data files have been provided:

*Data availability* (described in the omonym section): All raw data included in this study are available at gene expression omnibus (GEO). For 3-CePs, raw RNA-seq data and count matrix are under the GEO accession number GSE179057. Raw ATAC-seq data and peak matrix are available under the accession number GSE179059. Both datasets are collected in a GEO SuperSeries (GSE179064). Cisplatin RNA-seq data and count matrix are available at the accession number GSE207611, the raw ATAC-seq data and peak matrix are available under the number GSE207607. Both are collected in a GEO SuperSeries (GSE207612).

The cell line expression data employed in the prediction pipeline were downloaded from https://www.proteinatlas.org/about/download. The file *RNA HPA cell line gene data* contains transcript expression levels summarized per gene in 69 cell lines and is based on the Human Protein Atlas version 20.0 and Ensembl version 92.38.

Similarly, the TCGA expression data from cancer cell samples (the Cancer Genome Atlas) were downloaded from the same web page of the Human Cell Atlas (Transcript expression levels summarized per gene in 7932 samples from 17 different cancer types). Data are based on The Human Protein Atlas version 20.0 and Ensembl version 92.38.

Cisplatin IC50s in different cell lines can be accessed through the Genomics of Drug Sensitivity in Cancer database (https://www.cancerrxgene.org/compound/Cisplatin/1005/overview/ic50) (Yang et al., 2013). Codes to reproduce both pre-processing and downstream analyses reported in this manuscript are available at the public repository <https://github.com/ccarraro/3-CePs_prediction>. The hCoCena script is accessible at <https://github.com/MarieOestreich/hCoCena> (vertical integration code available upon request).

*Supplementary Data* are available as .xlsx or .pdf files.
